# Evaluation of a Supervised Adapted Physical Activity Program Associated or Not with Oral Supplementation with Arginine and Leucine in Subjects with Obesity and Metabolic Syndrome: A Randomized Controlled Trial

**DOI:** 10.3390/nu14183708

**Published:** 2022-09-08

**Authors:** Vanessa Folope, Caroline Meret, Ingrid Castres, Claire Tourny, Estelle Houivet, Sébastien Grigioni, Hélène Lelandais, André Petit, Aude Coquard, Charlène Guérin, Muriel Quillard, Christine Bôle-Feysot, Pierre Déchelotte, Najate Achamrah, Moïse Coëffier

**Affiliations:** 1 Department of Nutrition and Specialized Centre for Obesity, Rouen University Hospital, CHU Rouen, 76031 Rouen, France; 2 Université de Rouen Normandie, Inserm UMR1073 “Nutrition, Inflammation and Microbiota-Gut-Brain Axis”, 76183 Rouen, France; 3 Clinical Investigation Centre CIC 1404—Biological Resources Centre, Inserm, Rouen University Hospital, CHU Rouen, 76031 Rouen, France; 4 Université de Rouen Normandie, CETAPS UR3832 “Research Center for Sports and Athletic Activities Transformations”, 76000 Rouen, France; 5 Department of Biostatistics, Rouen University Hospital, CHU Rouen, 76031 Rouen, France; 6 Department of Pharmacy, Rouen University Hospital, CHU Rouen, 76031 Rouen, France; 7 Department of Biochemistry, Rouen University Hospital, CHU Rouen, 76031 Rouen, France

**Keywords:** obesity, metabolic syndrome, adapted physical activity, arginine, leucine

## Abstract

Background: In patients with obesity and metabolic syndrome (MetS), lifestyle interventions combining diet, in particular, and physical exercise are recommended as the first line treatment. Previous studies have suggested that leucine or arginine supplementation may have beneficial effects on the body composition or insulin sensitivity and endothelial function, respectively. We thus conducted a randomized controlled study to evaluate the effects of a supervised adapted physical activity program associated or not with oral supplementation with leucine and arginine in MetS-complicated patients with obesity. Methods: Seventy-nine patients with obesity and MetS were randomized in four groups: patients receiving arginine and leucine supplementation (ALs group, n = 20), patients on a supervised adapted physical activity program (APA group, n = 20), patients combining ALs and APA (ALs+APA group, n = 20), and a control group (n = 19). After the baseline evaluation (m0), patients received ALs and/or followed the APA program for 6 months (m6). Body composition, MetS parameters, lipid and glucose metabolism markers, inflammatory markers, and a cardiopulmonary exercise test (CPET) were assessed at m0, m6, and after a 3-month wash-out period (m9). Results: After 6 months of intervention, we did not observe variable changes in body weight, body composition, lipid and glucose metabolism markers, inflammatory parameters, or quality of life scores between the four groups. However, during the CPET, the maximal power (Pmax and Ppeak), power, and O_2_ consumption at the ventilatory threshold (P(VT) and O_2_(VT)) were improved in the APA and ALs+APA groups (*p* < 0.05), as well as the forced vital capacity (FVC). Between m6 and m9, a gain in fat mass was only observed in patients in the APA and ALs+APA groups. Conclusion: In our randomized controlled trial, arginine and leucine supplementation failed to improve MetS in patients with obesity, as did the supervised adapted physical activity program and the combination of both. Only the cardiorespiratory parameters were improved by exercise training.

## 1. Introduction

Obesity, defined by the World Health Organization (WHO) as an increase in the adipose tissue with negative impacts on organisms, is a serious public health challenge, especially due to the huge growth of class II and III obesity. By 2050, obesity is expected to affect up to 20% of the world’s population [1,2]. Obesity is associated with increased cardiometabolic risks, particularly with increased visceral adipose tissue. Indeed, abdominal obesity is often associated with dyslipidemia, hypertension, hyperinsulinemia, and glucose intolerance [3]. The accumulation of visceral adipose tissue leads to an increase in the production of free fatty acids (FFA) and decreases the sensitivity of the insulin receptors [4]. The long-term consequence of this condition is an increase in pancreatic insulin secretion, then causing a rise in blood sugar levels (with the appearance of insulin resistance and risk of type-II diabetes), but also an increase in the level of triglycerides, all elements contributing to cardiovascular morbidity and mortality. The combination of these disturbances corresponds to metabolic syndrome (MetS). In 2005, the International Diabetes Federation defined abdominal obesity as a mandatory criterion for MetS, associated with two elements among the following: hypertriglyceridemia, low HDL cholesterol, hypertension (>130/85 mmHg), and hyperglycemia, or proven type-II diabetes. Obesity is also associated with other comorbidities, such as osteoarthritis and joint pathologies, the increased incidence of several cancers, and respiratory pathologies (i.e., obstructive sleep apnea) [3], but also psychological problems and reduced quality of life [5,6]. Therefore, there appears to be an essential need to prevent and treat obesity.

Lifestyle interventions combining, in particular, diet and physical exercise are recommended as the first line treatment. It is well established that aerobic physical training increases energy expenditure and activates lipolysis [7]. A physical activity program carried out for twelve weeks at the rate of five sessions per week and at an intensity of 60 to 85% of the maximum heart rate (HRmax) enables weight loss and a decrease in the total fat mass, as well as the visceral and subcutaneous fat mass in subjects with obesity [8]. However, it seems that this type of exercise does not induce any significant change in muscle mass. Thus, Hills et al. combined aerobic work with muscle strengthening and showed positive results [9]. However, a recent meta-analysis concluded that there is low to moderate evidence that a supervised adapted physical activity program improves multiple risk factors of MetS [10]. In particular, a high variability was observed among clinical studies [11,12,13,14,15]. High-protein diets with reduced carbohydrate consumption are frequently associated with physical activity programs. Reducing the carbohydrate/protein ratio induces the loss of fat mass [16] and contributes to the maintenance of muscle mass [17]. In order to optimize the impact of the high-protein diet, different nutrients and food supplements, such as essential fatty acids and amino acids, have been tested.

It has been reported that leucine is able to stimulate and regulate protein synthesis in the body [18], especially in the muscles [19]. In addition, the combination of calorie restriction with leucine supplementation in athletes resulted in a greater reduction in fat mass [20]. Leucine also has effects on the insulin signaling pathways and glucose metabolism by promoting glucose utilization. Interestingly, subjects with obesity have a reduced leucine level [20], which could partly contribute to the insulin resistance. In animal models, beta-Hydroxy-Beta-Methylbutyrate (HMB), a metabolite of leucine, improved fat utilization and increased fatty acid oxidation in adipocytes and muscle cells [21,22]. In humans, HMB in combination with resistance exercise resulted in lower values of fat mass [23]. Arginine, a conditionally essential amino, is well known for its role as a key substrate in the biosynthesis of nitric oxide (NO), involved in endothelial function. Lucotti et al. [24] showed that chronic L-arginine supplementation for 21 days improved the insulin sensitivity and endothelial function in 33 type-2 diabetic patients with obesity. Arginine supplementation (at 14%), combined with a mixture of other amino acids, including glutamine and branched-chain amino acids, also improved athletic performance [25]. Finally, arginine limited the increase in visceral fat in rats fed with a high-fat diet [26], and a program of 3-month arginine supplementation improved insulin sensitivity in humans [27]. Based on these data, the combination of specific amino acids such as arginine and leucine could thus be of interest in efforts to enhance the effects of physical training on the body composition, insulin resistance, or on inflammatory response in subjects with obesity and MetS.

Therefore, we aimed to evaluate the effects of adapted physical activity associated or not with arginine and leucine supplementation in subjects with obesity and MetS in a randomized controlled clinical trial.

## 2. Methods

The present study was an open randomized controlled study (ClinicalTrials Identifier: NCT01326416) supported by the French National Program for Clinical Research (PHRC n° 2008/066/HP), with an enrolment period spanning from 2011 to 2014. The study was approved by the ethics committee (CPP Sud Mediterranee). 

### 2.1. Subjects

The study was proposed to all patients with obesity and MetS reviewed at the Nutrition Department of the Rouen University Hospital according to the following inclusion/exclusion criteria. The inclusion criteria were: age from 18 to 55 years, a body mass index (BMI) greater than or equal to 30 kg/m^2^, and a MetS diagnosis according to the International Diabetes Federation. The exclusion criteria were: body weight greater than 135 kg for technical reasons (limits of the equipment), pulmonary disease, musculoskeletal abnormalities, tobacco consumption greater than or equal to eight cigarettes per day, treatment with beta-blockers, and pregnancy.

### 2.2. Trial Design

After checking the inclusion and exclusion criteria, the clinical trial was explained to all participants, who gave written informed consent. Patients were randomly assigned in a 1:1:1:1 ratio to one of the four following groups: the control group, arginine- and leucine-supplemented group (ALs), adapted physical activity group (APA), or the group treated with both arginine and leucine supplementation and adapted physical activity (ALs+APA). A block randomization was used and performed by the hospital pharmacy. After randomization, all the patients were followed monthly during the 6-month treatment period (from 0 (m0) to 6 months (m6)) and then assessed at 9 months (m9, after the 3-month wash-out period). Patients in the control group received routine clinical care with monthly visits to dietician. In the ALs group, from m0 to m6, patients received an oral supplementation with 9 g of arginine and 21 g of leucine per day. In the APA group, from m0 to m6, patients followed a program of physical activity 3 times per week. In the ALs+APA group, from m0 to m6, patients received 9 g arginine and 21 g leucine supplementation per day and followed the APA program 3 times per week. 

The collected and measured parameters are detailed in Supplemental Table S1. Briefly, at each visit (m0, m1, m2, m3, m4, m5, m6, and m9), the body weight, height, waist and hip circumferences, and bioimpedance-derived body composition were measured, and patients met with a dietician. In addition, at m0, m3, m6, and m9, indirect calorimetry and blood samples were performed. At m0, m6, and m9, patients completed a quality of life questionnaire, and their body composition was measured by dual X-ray absorptiometry (DXA, Lunar Prodigy Advance, GE healthcare). Finally, at m0 and m6, a cardiopulmonary exercise test (CPET) was also performed.

### 2.3. Amino Acid Supplementation

The arginine and leucine powders (Cooper, Melun, France) were prepared by the pharmacy of Rouen University Hospital and supplied to the patients for 1 month. Patients were instructed to consume 21 g of leucine and 9 g of arginine per day in 3 meals. At each visit, patients received new bottles for the next month. The chosen doses of arginine and leucine were based on a compromise between previous studies [21,22,23,24,25,26,27] and the most optimal doses for subjects with obesity, as this was nearly 2 and 3 times the amounts recommended for the daily intake of each product. 

### 2.4. Adapted Physical Activity

Patients from the APA and ALs+APA groups performed a 6-month structured adapted physical activity (APA) program under the supervision of sport science graduates at a rate of 3 days per week. Each session comprised of a warmup (10 min), endurance training (20–60 min), strength training (20 min), and a final stretching phase (10 min). The endurance training intensity was maintained at 60% of the heart rate reserve (HRR) ±10 beats/min, with the HRR estimated using the Karvonen equation (220–age–resting heart rate) [28]. The strength training consisted of 6 to 10 exercises without a load for all muscle groups. In accordance with physical recommendations, the subjects performed 1–3 sets of 8–12 repetitions of each exercise. The rest time was 1 min 30 s between each set and 3 min between each exercise.

### 2.5. Anthropometric Measures, Body Composition, and Resting Energy Expenditure Assessments

After overnight fasting, anthropometric measures were performed, including body weight (kg), body height (cm), and the waist and hip circumferences (cm). The waist circumference was measured at the mid-point between the bottom of the rib cage and the iliac crest, and the hip circumference was measured at the widest point at the hip. Body mass index was calculated as body mass divided by the square body height (Kg.m^−2^)

Then, bioimpedance (BIA) was performed in the supine position after 10 min of equilibration with Bodystat Quadscan 4000 equipement (BodyStat), as previously described [29]. A manufacturer-protected algorithm was used to calculate the fat mass and fat free mass. At m0, m6, and m9, dual X-ray absorptiometry (DXA, Lunar Prodigy Advance, GE healthcare) was also performed to measure the lean mass, fat mass, and abdominal fat mass, as well as indirect calorimetry (Quark RMR, Cosmed) to calculate the resting energy expenditure (REE, in kcal/day), as previously described [30,31]. 

### 2.6. Quality of Life Questionnaire

At m0, m6, and m9, patients filled in a quality-of-life questionnaire based on the Medical Outcome Study Short Form-36 (MOS SF-36) [32]. This internationally validated instrument for general health is often used in obesity research [33]. The SF-36 contains 36 questions covering functional health status and general health. The questions are categorized into eight subscales measuring physical functioning, role physical and bodily pain, general health, vitality, social function, and role emotional and mental health [34]. The first three subscales (i.e., physical functioning and role physical and bodily pain) reflect the physical component of health. Social functioning and role emotional and mental health cover the psychosocial aspect. Vitality and general health give an overall score of subjective health. A score between 0 and 100 was computed for each subscale, with 100 representing the most favorable state of health. From the subscales, two summary scores were calculated: the physical composite summary (PCS) and the mental composite summary (MCS).

### 2.7. Cardiopulmonary Exercise Test (CPET)

The CPET was performed using an electromagnetically braked cycle ergometer (BV Lode, Groningen, Netherlands), as previously reported [35]. Briefly, following a 3 min warmup and a progressive increase in the power output, a pedaling rate of 50–60 rpm was maintained throughout the test. The subjects were instructed to achieve the highest possible power output, named Pmax. Expired air was continuously monitored through a breath-by-breath system (Ergocard, Medisoft, Sorinnes, Belgium) for the measurement of ventilation (VE), oxygen consumption (VO_2_), and the carbon dioxide output (VCO_2_), as well as the heart rate by a 12-lead electrocardiogram (Medcard, Medisoft). The ventilatory threshold (VT) was determined using the computerized V-slope method [36]. 

### 2.8. Blood Parameters

After overnight fasting, blood samples were obtained and then immediately transferred for the analysis of the clinical routine parameters (glucose, insulin, lipids, etc.) or sent to the Biological Resource Center for centrifugation (3000 rpm, 15 min at 4 °C). Plasma was then removed and aliquots were frozen at −80 °C until the time of analysis. Leptin, adiponectin, TNFα, IL-6, and CRP were then measured by Luminex technology (Bio-Techne, R&D systems, Abingdon, UK), following manufacturer’s recommendations, using Bioplex equipment (Biorad, Marnes-la-Coquette, France).

### 2.9. Statistical Analysis

To reach the primary endpoint, i.e., a 10% reduction in the fat mass percentage measured by DXA between m0 and m6 in the ALs+APA group compared to a 2% change in the control group and a 6% change in the ALs or APA groups, we estimated that a sample size of 13 patients in each group (a total of 52 patients), at 80% power with an alpha value of 5%, would be sufficient. We thus decided to enroll 20 patients per group.

The patients’ characteristics were described using the median, first, and third quartiles [Q1; Q3] for the quantitative variables and absolute numbers with their percentages for the categorical variables. To compare the patients’ characteristics at the baseline and changes between m0 and m6 or between m6 and m9, Kruskal–Wallis tests were performed followed by Dunn’s post tests for continued data if necessary. Chi squared tests or Fisher’s exact tests were used for the discontinued data. A *p*-value of <0.05 was considered statistically significant. The statistical analysis was performed using SAS software (9.3 version, SAS Institute, Cary, NC, USA).

## 3. Results

### 3.1. At Baseline

**Figure 1:**
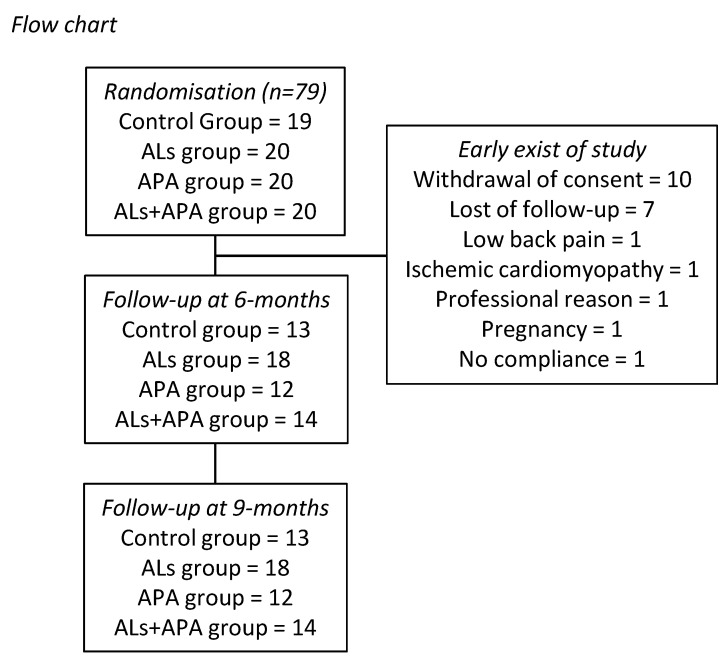
Flow chart. ALs, arginine and leucine supplementation; APA, adapted physical activity.

As described in the flow chart (Figure 1), we enrolled 19, 20, 20, and 20 patients in the control, ALs, APA, and ALS+APA groups, respectively. Patients’ characteristics at baseline are displayed in Table 1 and Supplemental Table S1. We did not observe differences between the groups except for the waist circumference (*p* = 0.0325, Kruskal–Wallis test). The waist circumference seemed to be higher in the control group, but the Dunn post test was not significant. A trend of higher body weight and BMI was also observed, but difference did not reach significance (*p* = 0.0572 and *p* = 0.0647, respectively, Table 1). The median age ranged from 39 to 44 years. The enrolled patients were mainly females (from 60 to 75% in each group). The median BMI ranged from 35.8 to 41 kg.m^−2^ and the median fat mass percentage from 41.6 to 47.3 (Table 1). 

### 3.2. At the End of the Program

After the 6-month program, data from 13, 18, 12, and 14 patients were available in the control, ALs, APA, and ALS+APA groups, respectively (Figure 1). The most frequent causes of early exit were the withdrawal of consent (n = 10) and loss of follow-up (n = 7). Due to this high rate of loss of follow-up, a per-protocol statistical analysis was performed.

After 6 months of treatment, the primary endpoint results were not significantly different between the different groups. Indeed, the fat mass changes measured by DXA were −2.10 [−3.68; 0.00], −2.22 [−5.90; −0.43], −2.60 [−4.40; −0.35], and −3.02 [−4.97; −1.40]% in the control, ALs, APA and ALS+APA groups, respectively, (*p* = 0.6440). Concerning secondary the endpoints, we did not observe differences in body weight, BMI, waist circumference, or body composition parameters (Table 2). In addition, biological parameter changes reflecting the lipid or glucose metabolism were not significantly different between the four groups, nor were the markers of hepatic function or inflammation (Table 3). Similarly, the changes in the quality-of-life scores (SF36 questionnaires) were not significantly different (Supplemental Table S2), even though we observed a trend of the improved psychosocial component in the intervention groups and particularly in the groups on the APA (Supplemented Table S2, *p* = 0.0819). By contrast, there was no variation in the SF36 component evaluating physical function between groups.

**Figure 2:**
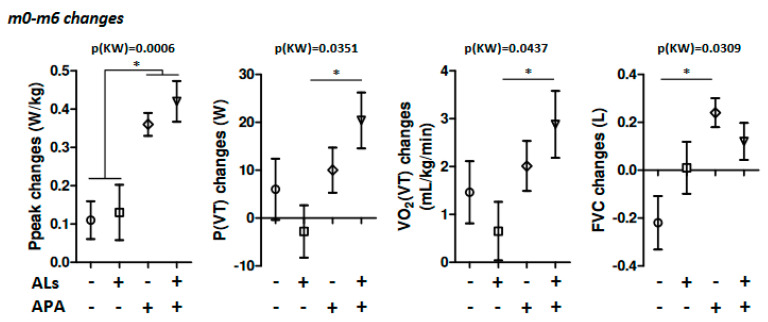
Changes after 6 months of treatment. Changes in the maximal power (Ppeak), power at ventilator threshold (P(VT)), O_2_ consumption at VT, and forced ventilator capacity (FVC) observed after 6 months of treatment in the 4 groups of patients receiving (or not) arginine and leucine supplementation (ALs) and following (or not) the adapted physical activity program (APA). Data were compared by the Kruskal–Wallis test (KW) followed by Dunn post tests. * *p* < 0.05.

However, we observed that the CPET exhibited modified results between the four groups (Table 4). Indeed, as described in Figure 2, the maximal power (Pmax) was increased more in the APA and ALs+APA groups compared to control and ALs groups (*p* = 0.0005). Similarly, Ppeak changes were also more pronounced in patients after they completed the APA program (*p* = 0.0006). The power at threshold (P(VT)) and O_2_ consumption were also affected. The forced vital capacity (FCV) or O_2_ consumption also seemed to increase more in patients from the APA and ALs+APA groups. 

### 3.3. Post-Test (+3 Months)

**Figure 3:**
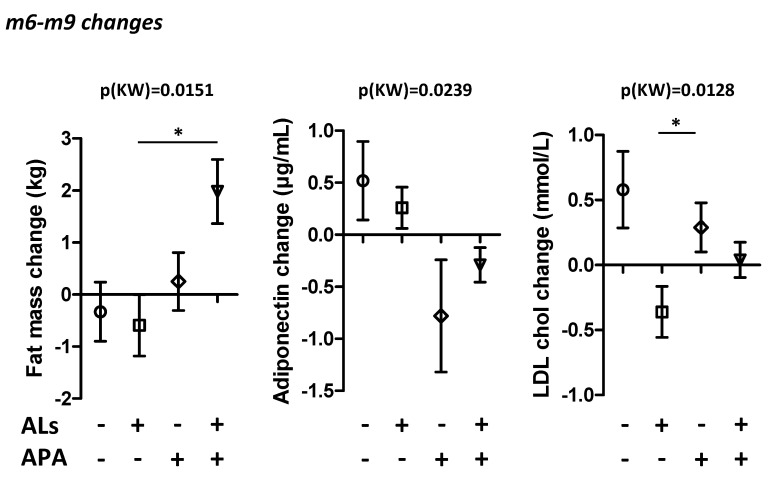
Changes after the 3-month wash-out period. Changes in fat mass, plasma adiponectin, LDL cholesterol observed after 3 months of the wash-out period following 6 months of treatment in the 4 groups of patients receiving (or not) arginine and leucine supplementation (ALs) and following (or not) the adapted physical activity program (APA). Data were compared using the Kruskal–Wallis test (KW) followed by Dunn post tests. * *p* < 0.05.

After 3 months of the wash-out period (m6 to m9), body weight and BMI changes were not different between the four groups (Supplemental Table S3). However, we observed significant differences in the fat mass changes between m6 and m9 (*p* = 0.0151). Indeed, patients in the ALs+APA group seemed to gain more fat mass compared to the other groups (Figure 3). Patients who followed an APA program exhibited a gain in fat mass, whereas patients in the control and ALs groups mainly exhibited a loss of fat mass. Changes in the lipid and glucose metabolism markers were not different between the groups, except for the LDL cholesterol level, which was more reduced in patients from ALs group (Figure 3). In addition, the plasma adiponectin levels were increased more in patients from the control and ALs groups and decreased in patients from the APA and ALs+APA groups (Figure 3). 

## 4. Discussion

In our randomized controlled study of patients with obesity, we failed to demonstrate beneficial effects of the 6-month program consisting of arginine and leucine supplementation in a supervised adapted physical activity program, neither in terms of body weight, body fat mass, nor the MetS parameters. However, we observed that exercise training was able to improve several cardiorespiratory variables, but it was also associated with an adiposity rebound 3 months after the end of the program. 

In patients with obesity, respiratory impairment, such as the impairment of the forced vital capacity, forced expiratory volume in one second, or expiratory reserve volume, has been reported [37,38]. Weight gain is associated with a decrease in the forced vital capacity and forced expiratory volume due to the effects of adiposity on chest wall compliance or inactivity [39]. A 12-week program combining strength and aerobic exercises improved dynamic cardiorespiratory changes in women with mainly class I and II obesity [40]. In the present study, improvements in several cardiopulmonary variables were also observed in patients following the 6-month physical activity program. Indeed, the VO_2_ at ventilatory threshold and forced vital capacity were improved after exercise training (by approximately 5 and 20%, respectively) in our population of subjects with class II and III MetS-complicated obesity. However, the VO_2_ max and expiratory volumes were not affected. Previous studies showed different levels of improvement in the VO_2_ max among women with obesity according to the duration and intensity of exercise, with values from 7.1% to 19% [40,41,42]. We also observed that the power (Pmax and Ppeak) was more markedly increased in patients after exercise training. Accordingly, in an open trial, a 12-week exercise program improved the Ppeak values of post-menopausal women with obesity and MetS [43]. All these data suggest that the 6-month supervised physical activity program combining endurance and resistance training was able to improve the cardiopulmonary variables in subjects with obesity and MetS. However, arginine and leucine supplementation alone or in combination with training had no effect on these parameters. 

In our clinical trial, the improvements in the cardiorespiratory parameters after the 6-month activity program were not associated with modifications in body weight or body composition. In a recent systematic review, Zouhal et al. [44] showed that aerobic and anaerobic training are often associated with improvements in the BMI and body fat content, except in one study performed on older women with obesity [45]. However, most of these studies were performed on children, adolescents, or metabolically heathy subjects with obesity. In metabolically unhealthy subjects with obesity, a one-year program combining Mediterranean diet nutritional counselling and high-intensity interval training (HIIT) improved the body composition, fasting glycaemia, insulin sensitivity, VO2 peak, and muscle endurance [46]. In addition, the effects of different intensities of resistance and endurance training have been evaluated: the higher the intensity of the training is, the greater the beneficial effects are [47]. One possible explanation for the absence of effects of our training program on the body weight and body composition could be the intensity of exercise, which was, however, sufficient for improving the cardiorespiratory parameters. In addition, we observed a body fat rebound 3 months after the end of the supervised training program, associated with a decrease in the plasma adiponectin levels. These data are in accordance with studies identifying weight regain upon medium- to long-term follow-up [48]. This phenomenon is likely multifactorial and can be explained by poor compliance or by behavioral or physiological adaptations. 

Finally, in our study, not only did the physical activity program fail to improve the body weight, body composition, and metabolic parameters, but the amino acids supplementation failed to do so as well. Previous studies suggested that branched amino acids are able to stimulate muscle protein synthesis and, thus, contribute to maintaining or increasing the lean mass [49]. In diet-induced obese mice, the simultaneous interventions of exercise and leucine increased the systemic insulin sensitivity by reducing the liver and adipose tissue inflammation and increasing the expression of adiponectin in the adipose tissue [50]. However, in accordance with our results, a recent study showed that branched-chain amino acids failed to augment the beneficial effects of resistance training in postmenopausal women [51]. To better understand the reasons behind the lack of amino acid effects, it would be interesting to take measurements of the plasma amino acids as a marker of amino acid absorption. However, as observed in older subjects [52], we cannot exclude alterations in splanchnic extraction in subjects with obesity that may limit the peripheral actions of supplemented amino acids. This point should be investigated further.

Our study has some limitations. The numbers of analyzed data for each group were small. Indeed, 22 out of 79 patients discontinued their participation in the program during the 6-month intervention period, which limits the strength of our results and underlines the poor compliance of the patients. A better selection of patients would have enhanced the level of compliance; however, it would have be less relevant to clinical practice. Consequently, we performed a per-protocol analysis of the data that constitutes another limitation of the present study. In addition, we did not make adjustments based on body weight, age, or sex, for instance, because these parameters were not different between the groups at baseline and because our study failed to show changes by the primary endpoint at 6 month. Finally, to better understand the observed results, it would be interesting to assess the feeding behavior and food intake of the patients, as well as their levels of previous training. Unfortunately, we did not have these data in the present study. 

## 5. Conclusions

In our randomized clinical trial, a 6-month supervised adapted physical activity program was associated with improvements in the cardiorespiratory capacities of MetS-complicated patients with obesity but failed to improve their body weight and body composition. Arginine and leucine supplementation had no effect. Our study underlines the need for randomized clinical trials evaluating the effects of multidisciplinary ambulatory intervention programs combining adapted physical activity and nutritional interventions, as well as psychosocial approaches in order to improve the care of patients with obesity and MetS. 

## Figures and Tables

**Table 1:** Demographic data at baseline.

	Control (n = 19)	ALs (n = 20)	APA (n = 20)	Als + APA (n = 20)	*p* Values
**Age (years)**	40 [36; 45]	39 [32; 44]	44 [34.5; 49.5]	44 [40; 52]	0.2323
**Females (n/%)**	13 (68.4)	12 (60)	14 (70)	15 (75)	0.7822
**Body weight (kg)**	115.6 [110.4; 124.5]	105.7 [95.9; 122.9]	103.4 [87.1; 117.1]	109.8 [89.1; 115.8]	0.0572
**BMI (kg.m^−2^)**	41 [35.8; 46.5]	38.2 [35.2; 41.1]	35.8 [32.5; 41]	36.9 [34.5; 39.2]	0.0647
**Waist circumference (cm)**	122 [117; 133]	118 [111.8; 127.5]	113 [111; 123]	114.8 [110; 121]	**0.0325**
**Hip circumference (cm)**	133 [111; 147]	121 [113; 128.5]	117 [109; 126]	124 [115; 130]	0.2182
**Waist/hip ratio**	0.97 [0.89; 1.02]	0.98 [0.93; 1.05]	1 [0.93; 1.02]	0.94 [0.91; 1.03]	0.5924
**Fat mass (kg)**	55.4 [33.1; 62.9]	43.6 [37.6; 48.1]	36.8 [30.7; 51.6]	40.7 [36.1; 50.8]	0.2539
**Fat mass (%)**	47.3 [30.8; 51.1]	41.6 [31.4; 48.6]	43.3 [27.3; 46.5]	42.8 [35.5; 46]	0.4951
**Fat free mass (kg)**	62 [58.3; 75.5]	62.1 [57.7; 74.2]	61.6 [52.3; 72.1]	62.5 [52.8; 67.7]	0.6649
**Fat free mass (%)**	52.7 [48.9; 69.2]	59 [51.8; 68.7]	56.8 [53.5; 72.8]	57.3 [54; 64.5]	0.4579
**Abdominal fat mass (kg)**	5.4 [4.2; 6.6]	5.1 [4.3; 6.5]	4.5 [3.8; 5]	4.8 [4.1; 5.8]	0.2820

Data are expressed as the median (quartiles 1 and 3). Continued data were compared using the Kruskal–Wallis test and discontinued data with the Khi2 or exact Fisher tests. BMI, body mass index.

**Table 2:** Demographic changes observed after 6 months of treatment: delta m0–m6.

	Control (n = 13)	ALs (n = 18)	APA (n = 12)	Als + APA (n = 14)	*p* Values
**Body weight (kg)**	−1.7 [−7.3; 0]	−3.65 [−7; 0]	−2.8 [−5.85; −1.3]	−4.05 [−6.2; −0.8]	0.9816
**BMI (kg.m^−2^)**	−0.64 [−2.41; −0.01]	−1.28 [−2.46; 0]	−0.91 [−2.1; −0.49]	−1.45 [−2.65; −0.3]	0.9887
**Waist circumference (cm)**	−5 [−10; −2]	−4 [−10; −3]	−5.5 [−10; −4]	−5 [−9; −3]	0.9340
**Hip circumference (cm)**	−6 [−11; −4]	−2 [−10; 0]	−1.5 [−9; 0]	−6 [−10; −3]	0.4373
**Waist/hip ratio**	0 [−0.02; 0.03]	−0.02 [−0.03; −0.01]	0 [−0.04; 0]	0.01 [−0.02; 0.03]	0.2758
**Fat mass (kg)**	−0.6 [−5.3; 0.3]	−0.9 [−4.7; −0.2]	−1.3 [−3.25; 0.2]	−3.15 [−6; −1.9]	0.4338
**Fat mass (%)**	−0.1 [−2.7; 1]	−0.95 [−5; 0.3]	−0.75 [−1.7; 0.25]	−2.7 [−3.3; −1]	0.2773
**Fat free mass (kg)**	−1.5 [−2.1; −0.2]	−0.6 [−3.2; 0.4]	−1.1 [−3.45; −0.05]	−1.05 [−1.4; 1.1]	0.8868
**Fat free mass (%)**	0.1 [−1; 2.7]	0.95 [−0.3; 5]	0.25 [0.05; 1.7]	2.7 [1; 3.3]	0.2638
**Abdominal fat mass (kg)**	−0.2 [−0.44; 0.24]	−0.2 [−1.2; 0]	−0.05 [−0.5; 0.18]	−0.7 [−0.9; −0.2]	0.1771

Data are expressed as median (quartiles 1 and 3). Continued data have were using the Kruskal–Wallis test and Dunn post tests. Values without a common letter significantly differ, *p* < 0.05. BMI, body mass index.

**Table 3:** Biological changes observed after 6 months of treatment: delta m0–m6.

	Control (n = 13)	ALs (n = 18)	APA (n = 12)	Als + APA (n = 14)	*p* Values
**Total cholesterol (mmol/L)**	−0.2 [−0.3; 0.3]	−0.05 [−0.5; 0.2]	−0.2 [−0.45; 0.25]	−0.1 [−0.3; 0.2]	0.9510
**HDL cholesterol (mmol/L)**	−0.03 [−0.15; 0.04]	0.02 [−0.12; 0.15]	0.09 [−0.03; 0.27]	0.06 [−0.02; 0.2]	0.2066
**LDL cholesterol (mmol/L)**	−0.11 [−0.57; 0.18]	0.05 [−0.49; 0.39]	−0.27 [−0.56; 0.04]	−0.14 [−0.26; 0.21]	0.5553
**Triglycerides (mmol/L)**	0.25 [−0.05; 0.36]	−0.29 [−0.66; 0.01]	−0.05 [−0.39; 0.17]	0.06 [−0.36; 0.47]	0.1939
**ASAT (UI/L)**	−2 [−7; 2]	−0.5 [−7; 4]	−1 [−4; 6]	0 [−3; 2]	0.6919
**ALAT (UI/L)**	−2 [−17; 1]	−0.5 [−18; 13]	−7 [−15; 3]	−4.5 [−12; 3]	0.6029
**ALP (UI/L)**	0 [−5; 3]	0 [−4; 7]	0 [−3; 4]	−3 [−9; 1]	0.5830
**γGT (UI/L)**	1 [−10; 4]	0 [−6; 7]	−5 [−9; 0]	−3 [−19; −1]	0.2764
**Fasting glycaemia (pmol/L)**	−0.3 [−0.7; −0.2]	0 [−0.4; 0.2]	−0.2 [−0.35; 0.1]	−0.1 [−0.3; 0.3]	0.1514
**Fasting insulin (mUI/L)**	−29 [−59; 7]	−17 [−53; 7]	−11 [−45; 50]	−23.5 [−44; −7]	0.6825
**HOMA-IR**	−7.86 [−21.04; 7.99]	−3.65 [−13.59; −0.6]	−3.05 [−10.8; 11.63]	−6.86 [−11.89; −1.71]	0.7646
**Leptin (ng/mL)**	−4.68 [−23.36; 15.19]	−10.45 [−26.53; 1.53]	−7.6 [−20.41; 10.86]	−10.36 [−33.72; −6.01]	0.5381
**Adiponectin (µg/mL)**	−0.42 [−0.99; 0.19]	−0.1 [−0.79; 1.11]	0.62 [−0.26; 1.7]	0.43 [−0.54; 0.74]	0.2557
**IL-6 (pg/mL)**	−0.21 [−0.32; 0.21]	−0.18 [−1; 0.32]	−0.21 [−0.36; 0.12]	−0.22 [−0.55; 0]	0.8735
**TNFα (pg/mL)**	0.09 [−0.3; 0.76]	−0.09 [−0.48; 0.34]	0.03 [−0.17; 0.78]	0.31 [0.09; 0.48]	0.3903
**CRP (mg/L)**	−0.73 [−1.45; 2.8]	−0.33 [−0.86; 0.35]	−0.14 [−0.43; 1.2]	−0.45 [−0.93; −0.01]	0.6269

Data are expressed as median (quartiles 1 and 3). Continued data were compared using Kruskal–Wallis and Dunn post tests. Values without a common letter significantly differ, *p* < 0.05.

**Table 4:** Changes in the CPET observed after 6 months of treatment: delta m0–m6.

	Control (n = 13)	ALs (n = 18)	APA (n = 12)	Als + APA (n = 14)	*p* Values
**Pmax (W)**	0 [0; 10] ^a^	7.5 [0; 15] ^a^	30 [20; 30] ^b^	25 [10; 40] ^b^	**0.0005**
**Ppeak (Pmax/poids) (W.kg^−1^)**	0.1 [0.03; 0.2] ^a^	0.2 [0; 0.3] ^a,b^	0.32 [0.3; 0.45] ^b,c^	0.4 [0.3; 0.6] ^c^	**0.0006**
**VO_2_ max (L.min^−1^)**	0.03 [−0.11; 0.1]	0.04 [−0.16; 0.17]	0.19 [0.02; 0.37]	0.27 [0.06; 0.42]	0.0708
**VO_2_ max (mL.kg^−1^.min^−1^)**	0.85 [−0.5; 3]	0.5 [−0.8; 1.7]	3.35 [1.15; 4.45]	4.1 [0.6; 6.4]	0.0965
**VCO_2_/VO_2_ max**	0.03 [−0.06; 0.1]	0.04 [−0.01; 0.09]	0 [−0.07; 0.07]	−0.01 [−0.1; 0.06]	0.3808
**Heart rate max (bpm)**	3 [−8; 11]	0.5 [−5; 6]	3 [−1; 7.5]	5 [−3; 13]	0.6059
**P(VT) (W)**	5 [−10; 20] ^a,b^	0 [−10; 10] ^a^	10 [0; 20] ^a,b^	20 [10; 30] ^b^	**0.0351**
**VO_2_ (VT) (mL.kg^−1^.min^−1^)**	2.3 [0.6; 2.5] ^a,b^	0.2 [−0.8; 1.6] ^a^	1.9 [0.6; 3.7] ^a,b^	2.45 [1.2; 3.8] ^b^	**0.0437**
**Heart rate (VT) (bpm)**	−1.0 [−8.0; 14.0]	−1.5 [−8.0; 8.0]	2.0 [−2.5; 7.0]	4.0 [−9.0; 20.0]	0.8214
**Slow VC (L)**	−0.23 [−0.45; 0.22]	−0.04 [−0.48; 0.28]	0.21 [−0.03; 0.35]	0.27 [−0.2; 0.5]	0.1091
**Forced VC (L)**	−0.19 [−0.63; 0.1] ^a^	0.04 [−0.24; 0.22] ^a,b^	0.22 [0.07; 0.41] ^b^	0.18 [−0.11; 0.38] ^a,b^	**0.0309**
**Inspiratory VC (L)**	−0.01 [−0.05; 0.11]	−0.1 [−0.26; 0.13]	−0.08 [−0.25; 0.02]	0.16 [−0.23; 0.3]	0.4904
**Expiratory reserve volume (L)**	−0.02 [−0.46; 0.15]	−0.02 [−0.29; 0.59]	0.35 [0.16; 0.62]	0.17 [−0.11; 0.46]	0.1554
**Forced expiratory volume (L)**	−0.17 [−0.57; 0.07]	0.01 [−0.30; 0.23]	0.12 [−0.07; 0.34]	0.12 [−0.07; 0.23]	0.1276

Data are expressed as median (quartiles 1 and 3). Continued data were compared with Kruskal–Wallis and Dunn post tests. P, power; VT, ventilator threshold; VC, vital capacity. Values without a common letter significantly differ, *p* < 0.05.

## Data Availability

Not applicable.

## Supplementary Materials

The following supporting information can be downloaded at: https://www.mdpi.com/article/10.3390/nu14183708/s1, Table S1: Biological data at baseline; Table S2: Other changes observed after 6 months of treatment: delta m0-m6; Table S3: Other changes observed between 6-months and 9 months of follow-up: delta m6-m9.
