# Supplementary material for: Evaluation of a Supervised Adapted Physical Activity Program Associated or Not with Oral Supplementation with Arginine and Leucine in Subjects with Obesity and Metabolic Syndrome: A Randomized Controlled Trial
Source: Nutrients. 2022 Sep 8;14(18):3708. doi: 10.3390/nu14183708 (PMC9501942; doi:10.3390/nu14183708)
Supplement: Supplementary file 1 [file nutrients-14-03708-s001.zip › nutrients-1852663-supplementary.pdf]

Evaluation of a supervised adapted physical activity program associated or not to oral supplementation with arginine and leucine in obese subjects with metabolic syndrome: a randomized controlled trial.

Vanessa Folope et al.

Supplemental materials

Supplemental Table S1: Biological data at baseline

	Control (n=19)	ALs (n=20)	APA (n=20)	Als + APA (n=20)	P values
**REE (kcal/day)**	1996 [1924 ; 2207]	1954.5 [1743 ; 2124]	1765.5 [1637 ; 2123]	1980 [1778 ; 2134]	0.2491
**Total cholesterol (mmol/L)**	5.3 [4.9 ; 6.8]	5.8 [5.2 ; 6.2]	5.6 [5 ; 6.4]	5.1 [4.4 ; 5.6]	0.2409
**HDL cholesterol (mmol/L)**	1.1 [0.9 ; 1.3]	1.1 [0.9 ; 1.2]	1.1 [0.9 ; 1.5]	1 [1 ; 1.1]	0.5624
**LDL cholesterol (mmol/L)**	3.3 [2.7 ; 4.6]	4.1 [3.1 ; 4.5]	3.7 [2.7 ; 4.2]	3.3 [2.6 ; 3.6]	0.3004
**Triglycerides (mmol/L)**	1.9 [1.3 ; 2.4]	1.7 [1.4 ; 2.4]	1.9 [1.5 ; 2.3]	1.5 [1.1 ; 2.1]	0.3745
**ASAT (UI/L)**	25 [20 ; 32]	27 [21.5 ; 34.5]	24.5 [21.5 ; 27]	22 [20.5 ; 25]	0.3379
**ALAT (UI/L)**	37 [25 ; 65]	49 [26 ; 59.5]	33 [27 ; 49]	33.5 [27.5 ; 40.5]	0.6148
**ALP (UI/L)**	69 [58 ; 76]	58.5 [47.5 ; 68]	74 [63.5 ; 82.5]	66 [53 ; 78]	0.0971
**γGT (UI/L)**	33 [24 ; 78]	39 [24 ; 50.5]	29 [20.5 ; 49.5]	29.5 [24.5 ; 60.5]	0.6706
**Fasting glycaemia (pmol/L)**	5.6 [5.1 ; 6.3]	5.1 [4.8 ; 6]	5.5 [5.1 ; 6.3]	5.5 [4.9 ; 5.9]	0.4529
**Fasting Insulin (mUI/L)**	155 [134 ; 219]	139.5 [97.5 ; 175.5]	111 [80 ; 201]	148.5 [107 ; 180]	0.2178
**HOMA-IR**	38.3 [33.2 ; 52.4]	30.6 [17 ; 46.2]	23.6 [19 ; 57.2]	34.7 [28.7 ; 43.3]	0.1805
**Leptin (ng/mL)**	73.3 [36.3 ; 136.1]	69.9 [42.6 ; 157.2]	84.1 [62.7 ; 132.9]	88.1 [48.6 ; 125.2]	0.9677
**Adiponectin (µg/mL)**	6.2 [4.5 ; 8.7]	5.4 [4.1 ; 7.4]	6.3 [4.9 ; 8.3]	5.1 [4.5 ; 8.3]	0.6804
**IL-6 (pg/mL)**	1.4 [1.3 ; 3.2]	1.4 [1.1 ; 2.5]	1.6 [1.2 ; 2.5]	1.5 [0.9 ; 1.9]	0.9589
**TNFα (pg/mL)**	1.5 [1.4 ; 1.7]	1.7 [1.2 ; 1.9]	1.7 [1.4 ; 2.1]	1.8 [1.4 ; 2.1]	0.5466
**CRP (mg/L)**	5.1 [2.1 ; 5.9]	3.5 [2.4 ; 5.8]	5.3 [2.8 ; 6.5]	3.4 [2.8 ; 5.3]	0.6361
**Pmax (W)**	140 [120 ; 180]	155 [130 ; 195]	140 [110 ; 180]	145 [120 ; 155]	0.6036
**Ppeak (Pmax/poids) (W.kg^{-1})**	1.2 [1 ; 1.8]	1.6 [1.3 ; 1.8]	1.5 [1.1 ; 1.7]	1.3 [1.2 ; 1.6]	0.3412
**VO_2 max (L.min^{-1})**	2.1 [1.9 ; 2.6]	2.2 [2 ; 2.6]	2.2 [1.6 ; 2.6]	1.9 [1.6 ; 2.4]	0.3245
**VO_2 max (mL.kg^{-1}.min^{-1})**	17.5 [16.4 ; 23.7]	21.4 [18.5 ; 24.1]	21.4 [17.4 ; 24.8]	20.4 [16.9 ; 21.2]	0.4278
**VCO_2/VO_2 max**	1.3 [1.3 ; 1.4]	1.3 [1.3 ; 1.4]	1.3 [1.3 ; 1.3]	1.3 [1.3 ; 1.4]	0.1773

(Supplemental Table S1 continued)

**Heart rate max (bpm)**	170 [161 ; 177]	172 [163 ; 179]	163.5 [147 ; 176]	157 [147 ; 169.5]	0.0851
**P(VT) (W)**	80 [70 ; 105]	90 [75 ; 120]	80 [70 ; 115]	80 [60 ; 100]	0.5218
**VO_2 (VT) ($mL.kg^{-1}.min^{-1}$)**	11.5 [9.3 ; 13.8]	13.6 [10.5 ; 15.6]	12 [9.8 ; 15.6]	12.1 [10.6 ; 12.8]	0.3729
**Heart rate (VT) (bpm)**	128 [120 ; 137]	131 [122 ; 138]	120 [110 ; 140]	125 [115 ; 134]	0.3686
**Slow VC (L)**	3.8 [3.4 ; 4.4]	4 [3.7 ; 4.9]	3.8 [3.4 ; 4.3]	3.9 [3.4 ; 4.9]	0.5657
**Forced VC (L)**	3.5 [3.1 ; 4.2]	4 [3.4 ; 4.6]	3.6 [3.1 ; 4.2]	3.5 [3.3 ; 4.4]	0.5520
**Inspiratory VC (L)**	3 [2.8 ; 3.4]	3.4 [2.9 ; 4.2]	3.4 [2.7 ; 3.7]	3.2 [2.7 ; 4]	0.3622
**Expiratory Reserve Volume (L)**	0.8 [0.5 ; 1.1]	0.6 [0.4 ; 0.9]	0.5 [0.2 ; 0.8]	0.6 [0.5 ; 0.9]	0.1931
**Forced expiratory volume (L)**	2.8 [2.5 ; 3.4]	3.2 [2.7 ; 3.5]	2.9 [2.5 ; 3.3]	2.7 [2.5 ; 3.3]	0.6392
**Dyspnoea (n/% of patients)**	15 (88.2)	19 (95)	17 (85)	17 (85)	0.7889
**Fatigue (n/% of patients)**	3 (17.6)	4 (20)	3 (15)	5 (25)	0.9239
**SF36 (physical function)**	213.5 [188 ; 276]	291 [249 ; 321]	262 [163 ; 318]	275 [208 ; 331.5]	0.2028
**SF36 (Mental Health)**	211 [161.3 ; 285]	273.5 [237.3 ; 330.8]	270 [156.9 ; 308.3]	247 [132.5 ; 298.5]	0.2892

Data are expressed as median (quartiles 1 and 3). Continued data have been compared with Kruskal-Wallis and discontinued data with Khi2 or exact Fisher tests. BMI, body mass index; REE, resting energy expenditure; P, power; VT, ventilator threshold; VC, vital capacity.

Supplemental Table S2: Other changes observed after 6 months of treatment: delta m0-m6.

	Control (n=13)	ALs (n=18)	APA (n=12)	Als + APA (n=14)	P values
**REE (kcal/day)**	-49 [-112 ; 6]	-93.5 [-188 ; 21]	-43 [-62 ; 5]	-49 [-163 ; 54]	0.8754
**SF36 (physical function)**	42 [-83 ; 112]	27 [-13 ; 51]	23 [1 ; 154]	78 [29 ; 118]	0.1456
**SF36 (Mental Health)**	3.5 [-89.3 ; 41.3]	17 [-6.5 ; 36.5]	75.8 [5 ; 107]	45.5 [19 ; 86.5]	0.0819

Data are expressed as median (quartiles 1 and 3). Continued data have been compared with Kruskal-Wallis and Dunn post-tests. BMI, body mass index; REE, resting energy expenditure; P, power; VT, ventilator threshold; VC, vital capacity.

Supplemental Table S3: Other changes observed between 6-months and 9 months of follow-up: delta m6-m9

	Control (n=13)	ALs (n=18)	APA (n=12)	Als + APA (n=14)	P values
**REE (kcal/day)**	-42 [-98 ; 39]	26 [-182 ; 208]	30.5 [-7 ; 103]	19 [-39 ; 57]	0.4793
**Total cholesterol (mmol/L)**	0.2 [-0.05 ; 0.45]	-0.05 [-0.4 ; 0.1]	0.3 [-0.1 ; 0.8]	0 [-0.18 ; 0.3]	0.2949
**HDL cholesterol (mmol/L)**	0.05 [-0.07 ; 0.14]	0.03 [-0.08 ; 0.18]	0.02 [-0.04 ; 0.07]	0.06 [-0.07 ; 0.19]	0.9440
**LDL cholesterol (mmol/L)**	0.32 [-0.06 ; 1.46] [a,b]	-0.24 [-0.46 ; 0.04] [a]	0.6 [-0.13 ; 0.72] [b]	0.08 [-0.44 ; 0.32] [a,b]	**0.0128***
**Triglycerides (mmol/L)**	-0.1 [-0.77 ; 0.23]	0.22 [-0.29 ; 1.09]	-0.18 [-0.63 ; 0.38]	-0.08 [-0.55 ; 0.15]	0.1316
**ASAT (UI/L)**	-0.5 [-2.5 ; 5]	0 [-1 ; 4]	-1 [-7 ; 4]	1 [-4 ; 2]	0.9139
**ALAT (UI/L)**	-2 [-4.5 ; 4.5]	-2.5 [-10 ; 8]	-3 [-10 ; 3]	-1 [-2 ; 5]	0.7071
**ALP (UI/L)**	-0.5 [-2.5 ; 2.5]	-0.5 [-4 ; 4]	1 [-8 ; 6]	1 [-2 ; 4]	0.9480
**γGT (UI/L)**	-0.5 [-5.5 ; 2]	1 [-3 ; 8]	0.5 [-2 ; 3]	-1 [-7 ; 5]	0.8531
**Fasting glycaemia (pmol/L)**	0.1 [-0.25 ; 0.55]	0 [-0.2 ; 0.4]	0.1 [0 ; 0.3]	0.05 [-0.2 ; 0.3]	0.8953
**Fasting Insulin (mUI/L)**	-0.5 [-40 ; 27.5]	1 [-4 ; 49]	0 [-22 ; 55]	-0.5 [-13 ; 33]	0.6731
**HOMA-IR**	1.03 [-7.05 ; 7.64]	0.76 [-0.96 ; 10.76]	0.28 [-4.95 ; 11.36]	0.86 [-3.94 ; 8.21]	0.8013
**Leptin (ng/mL)**	4.81 [-18.74 ; 22.84]	7.73 [-8.21 ; 17.32]	2.86 [-1.58 ; 20.17]	2.17 [-7.92 ; 17.7]	0.9246
**Adiponectin (µg/mL)**	0.36 [-0.39 ; 1.39]	0.12 [-0.3 ; 0.8]	-1.02 [-1.72 ; 0.08]	-0.42 [-0.56 ; 0.09]	**0.0239***
**IL-6 (pg/mL)**	-0.04 [-0.77 ; 0.11]	0.11 [-0.32 ; 0.33]	0.27 [0 ; 1.47]	0.48 [-0.21 ; 0.73]	0.1903
**TNFα (pg/mL)**	-0.4 [-0.6 ; 0.14]	0 [-0.24 ; 0.42]	-0.04 [-0.17 ; 0.11]	-0.41 [-0.57 ; 0.09]	0.2669
**CRP (mg/L)**	0.21 [-1.3 ; 1.05]	0.28 [-0.19 ; 1.35]	-0.1 [-0.29 ; 1.2]	0.29 [-0.46 ; 0.77]	0.8182
**SF36 (physical function)**	-15 [-35 ; 9]	3 [-30 ; 43]	-9.5 [-46 ; 19.5]	-7 [-18 ; 19]	0.8454
**SF36 (Mental Health)**	12.75 [-0.3 ; 23.7]	-8.5 [-40.5 ; 18.5]	0.65 [-68.75 ; 16.25]	8.25 [-102.3 ; 30.5]	0.4049

Data are expressed as median (quartiles 1 and 3). Continued data have been compared with Kruskal-Wallis and Dunn post-tests. BMI, body mass index; REE, resting energy expenditure. Values without a common letter significantly differ, *p<0.05.
